# An Interesting Presentation of Hypercalcaemia in a Middle-Aged Male

**DOI:** 10.7759/cureus.86682

**Published:** 2025-06-24

**Authors:** Aminah Patel, Rehan Siddique, Amber Khan

**Affiliations:** 1 Diabetes and Endocrinology, Salford Royal Hospital, Salford, GBR

**Keywords:** diagnosis, granuloma, hypercalcaemia, sarcoidosis, sjögren’s syndrome

## Abstract

A 41-year-old male presented to the Emergency Department with hypercalcaemia and acute kidney injury. He initially presented with bilateral parotid swelling which was treated as mumps. This was later investigated for suspected Sjögren’s syndrome, but further evaluation led to a diagnosis of sarcoidosis. Imaging confirmed extensive mediastinal and hilar lymphadenopathy, and a skin biopsy revealed non-caseating granulomas, confirming sarcoidosis. This case highlights the complexity of diagnosing hypercalcaemia and the importance of considering systemic granulomatous diseases like sarcoidosis in the differential diagnoses.

## Introduction

Hypercalcaemia is a common electrolyte disturbance, commonly associated with primary hyperparathyroidism and malignancy [1]. However, granulomatous diseases like sarcoidosis can also lead to hypercalcaemia [2]. The causes of hypercalcaemia can be broadly divided into parathyroid hormone (PTH)-mediated, calcitriol-mediated, malignancy-related and other rare mechanisms [3].

Sarcoidosis is a multisystem granulomatous disease of unknown aetiology, typically affecting adults aged 20-60 years, with variable prevalence based on ethnicity and geography [2,4]. Hypercalcaemia occurs in up to 10-20% of patients with sarcoidosis [2]. This is primarily due to increased extrarenal production of 1,25-dihydroxyvitamin D (calcitriol) by activated macrophages within granulomas, leading to increased intestinal calcium absorption [3,4]. Unlike PTH-driven hypercalcaemia, this mechanism is independent of feedback regulation, which can result in persistent elevation of calcium levels.

Clinically, hypercalcaemia can lead to nephrocalcinosis, acute kidney injury (AKI), neurocognitive symptoms, gastrointestinal disturbance, and cardiac arrhythmias, underscoring the importance of early recognition and management [1,3].

This case discusses a middle-aged male patient presenting with hypercalcaemia and acute kidney injury (AKI), initially suspected to have Sjögren’s syndrome based on clinical presentation, only to be later diagnosed with sarcoidosis. The diagnostic journey, differential considerations, and management strategies are outlined.

## Case presentation

A 41-year-old white Caucasian male initially presented to the Emergency Department (ED) with a two-week history of bilateral parotid gland swelling in May 2024. Initially, he was presumed to have mumps by his primary care doctor. The swelling became more substantial and painful leading to this presentation to the ED. The Ears, Nose, Throat (ENT) team reviewed the patient and discharged him with a one-week course of oral co-amoxiclav and follow-up for an outpatient ultrasound (US) of the parotid glands and the plan for a review in clinic.

**Figure 1:**
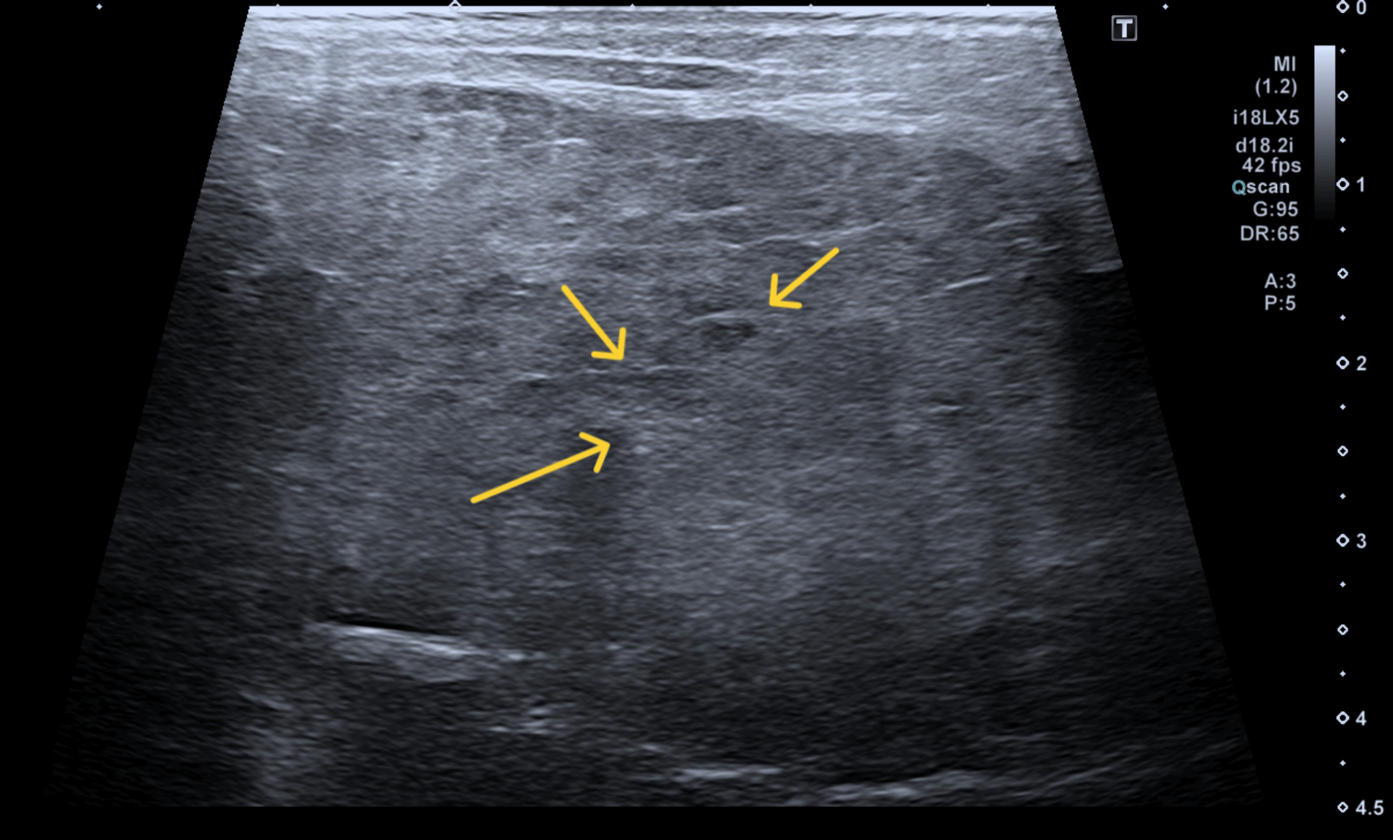
Arrows showing diffuse hypertrophy with patchy heterogenous echogenicity in parotid gland.

**Figure 2:**
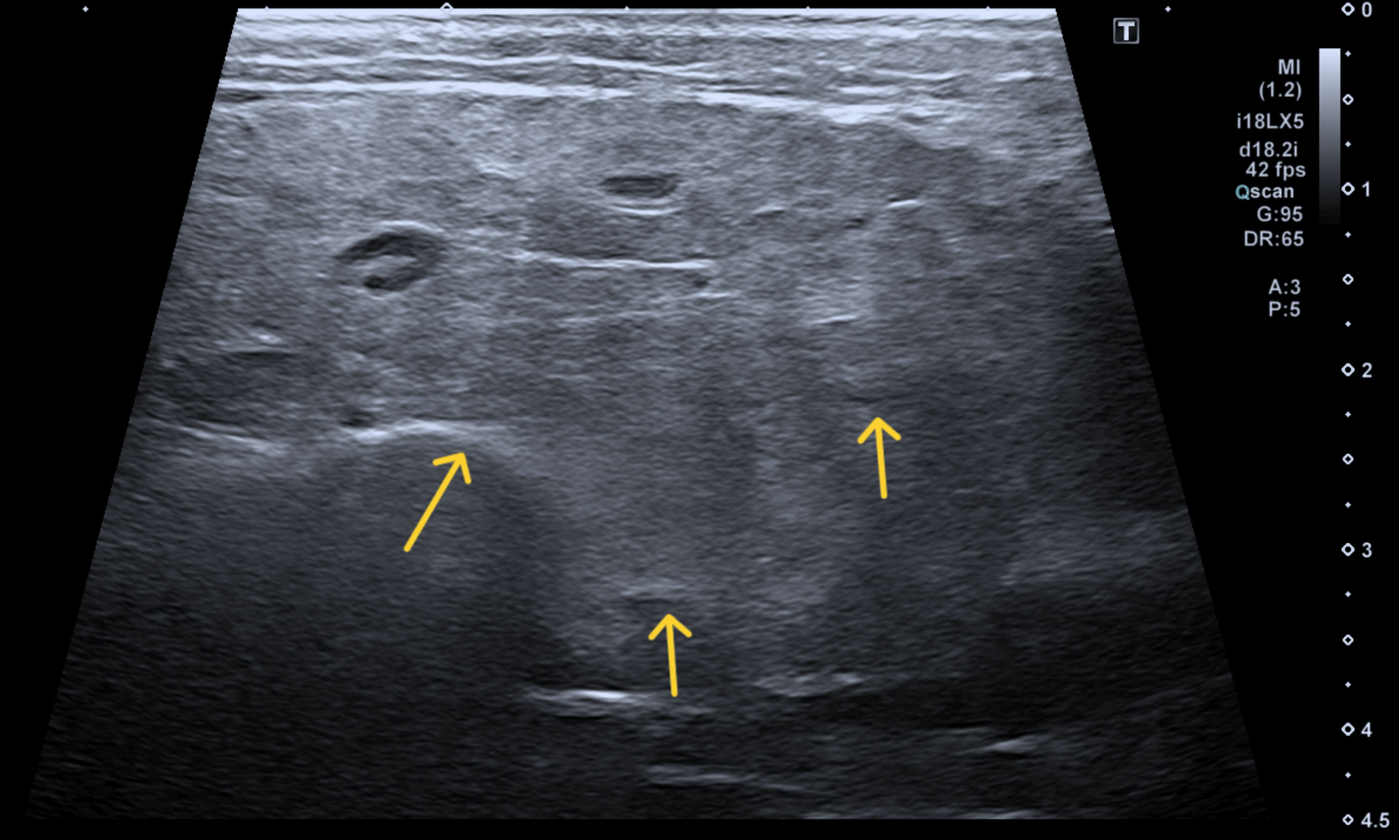
Arrows showing diffuse hypertrophy with patchy heterogenous echogenicity in submandibular gland.

In June, the patient underwent US parotid salivary glands which showed that there was diffuse hypertrophy with patchy heterogenous echogenicity in both parotid (Figure 1) and submandibular salivary glands (Figure 2), and echo textures were suspicious for Sjögren’s syndrome. Subsequently, the patient was seen in the ENT clinic in early August, where a punch biopsy of his inner lip was performed to support the diagnosis.

In the interim, the patient saw a rheumatologist. It was suggested that the presentation was less indicative of Sjögren’s syndrome and more in keeping with sarcoidosis based on the full history obtained by the rheumatologist. They recommended a series of initial blood tests. The following day, the patient was contacted and advised to go to A&E due to elevated calcium levels and significantly impaired kidney function. Consequently, the patient presented to A&E in mid-August with hypercalcaemia and stage 2 AKI.

The patient was admitted under the care of the medical team. He reported severe dry eyes and mouth, which began approximately three weeks after the initial parotid swelling. He had attempted various over-the-counter artificial saliva products without success. He also experienced profound fatigue and ongoing arthralgia, which had recently prevented him from working as a pipelayer. Over the past four months, he had lost approximately 25 kilograms due to a lack of appetite and dry mouth. Additionally, two weeks prior to presentation, he had developed a rash across his trunk and lower legs. He also reported bilateral hand paraesthesia, primarily affecting the ulnar digits. He did not have any urinary or bowel symptoms and denied any respiratory or cardiac symptoms.

His past medical history included hypertension which was well managed with 1.25 mg Ramipril. He was a lifelong non-smoker and previously consumed 36 units of alcohol per week, but discontinued drinking approximately three months prior to this current presentation due to feeling generally unwell. There was no family history of autoimmune conditions.

On examination, his vital signs were within normal limits. Respiratory, cardiac, abdominal and joint examinations were normal. Neurological examination was significant for reduced sensation in the ulnar distribution of both hands. Dermatological examination revealed a scaly non-tender, non-pruritic maculopapular rash on the lower shins and anterior thighs. There was a blanching nodulo-papular rash on the lower abdomen and lower back.

Investigations

His initial blood tests showed elevated corrected calcium level of 3.33 mmol/L (reference range: 2.20-2.60 mmol/L), normal phosphate of 1.33 mmol/L (reference range: 0.80-1.50 mmol/L), low PTH of <0.5 pmol/L (reference range: 2.0-9.3 pmol/L) and low vitamin D levels of 25.8 nmol/L (reference range: 50-125 nmol/L).

With regards to his kidney function he had a raised urea of 9.6 mmol/L (reference range: 2.5-7.8 mmol/L), rise in the creatinine of 194 umol/L (reference range: 65-104 umol/L), and a decline in his estimated Glomerular Filtration Rate (eGFR) which was 33 mL/min/1.73 m^2^ (reference range: >90 mL/min/1.73 m^2^), when it was 89 mL/min/1.73 m^2^, approximately three months previously. The laboratory results are summarised in Table 1.

**Table 1:** Blood tests on admission.

	Result	Reference Range
Sodium	137 mmol/L	133–146 mmol/L
Potassium	4.5 mmol/L	3.5–5.3 mmol/L
Urea	9.6 mmol/L	2.5–7.8 mmol/L
Creatinine	194 umol/L	65–104 umol/L
Corrected Calcium	3.33 mmol/L	2.20–2.60 mmol/L
Phosphate	1.33 mmol/L	0.80–1.50 mmol/L
Protein	70 g/L	61–78 g/L
Albumin	42 g/L	35–50 g/L
Alkaline Phosphatase	84 U/L	30–130 U/L
Total Bilirubin	10 umol/L	0–20 umol/L
Alanine transaminase	21 U/L	7–40 U/L
Estimated Glomerular Filtration Rate	33 mL/min/1.73 m^2^	>90 mL/min/1.73 m^2^
Ferritin	174 ug/l	22–322 ug/l
Vitamin B12	223 ng/L	211–911 ng/L
Folate	3.64 ug/l	>5.38 ug/l
Iron	15.4 umol/L	11.0–31 umol/L
Transferrin	2.41 g/L	2.15–3.65 g/L
Parathyroid Hormone	<0.5 pmol/L	2.0–9.3 pmol/L
25-Hydroxyvitamin D	25.8 nmol/L	50–125 nmol/L
C-reactive Protein	18 mg/L	<10.0 mg/L
Erythrocyte sedimentation rate	33 mm/hr	1–7 mm/hr
Glycated hemoglobin	36 mmol/mol	20–42 mmol/mol
Glucose	4.9 mmol/mol	3.6–5.3 mmol/mol
Haemoglobin	142 g/L	130–180 g/L
White Blood Cell	5.3 x 10^9^/L	4.0–11.0 x 10^9^/L
Neutrophil	3.6 x 10^9^/L	1.8–7.5 x 10^9^/L
Lymphocyte	0.7 x 10^9^/L	1.0–4.0 x 10^9^/L
Eosinophils	0.2 x 10^9^/L	0.0–0.4 x 10^9^/L
Basophils	0.1 x 10^9^/L	0.0–0.1 x 10^9^/L
Platelets	318 x 10^9^/L	150–450 x 10^9^/L
Hepatitis B Screen	Negative	
Hepatitis C Screen	Negative	
Human Immunodeficiency Virus Screen	Negative	
Epstein–Barr Virus Screen	Immunoglobulin G – Positive, Immunoglobulin M - Negative	Consistent with past EBV infection
Immunoglobulin G	12.20 g/L	6.50–16.00 g/L
Immunoglobulin A	3.77 g/L	0.40–3.50 g/L
Immunoglobulin M	0.95 g/L	0.50–3.00 g/L
Serum Angiotensin Converting Enzyme	<8 U/L	13.0–64.0 U/L

**Figure 3:**
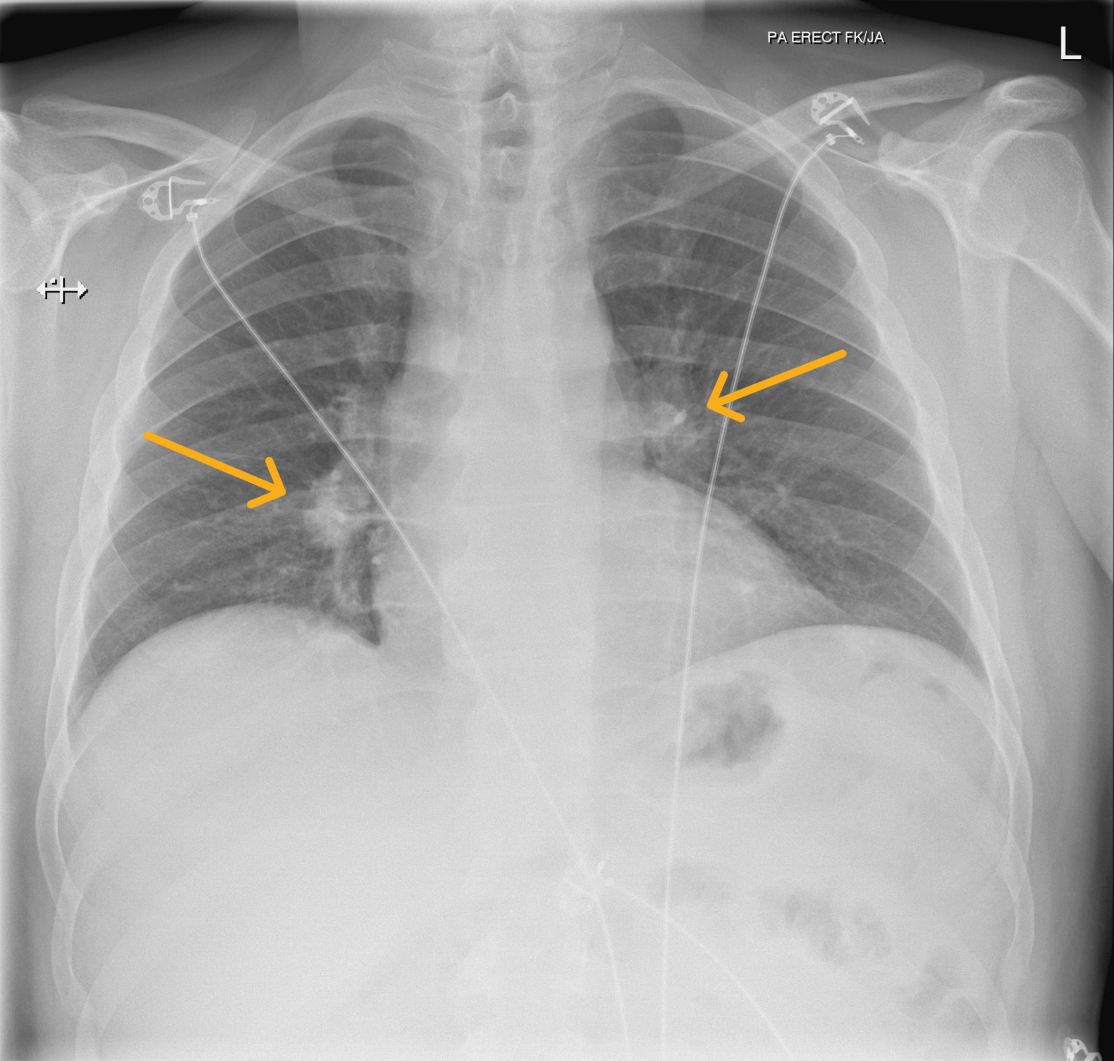
Chest X-ray with arrows showing symmetrical hilar and mediastinal lymphadenopathy.

Serum Angiotensin Converting Enzyme (ACE) was low with a value <8 U/L (13.0-64.0 U/L). An Electrocardiogram (ECG) showed sinus rhythm and normal QT interval. Chest X-ray revealed symmetrical hilar and mediastinal lymphadenopathy (Figure 3).

Management

The patient was commenced on intravenous fluids, approximately 45-66 mL/kg per day with daily monitoring of renal function and electrolytes. A Computed Tomography (CT) chest, abdomen, pelvis and neck was requested to rule out any underlying malignancy leading to high calcium and suppressed PTH.

Renal input was requested for evaluation of AKI and the need for renal biopsy. It was concluded by them to defer steroid initiation until further investigation results were available.

The patient was evaluated by dermatology and noted to have erythematous macules, subcutaneous nodules, and plaques on the bilateral thighs, as well as erythematous scaly macules and plaques on the bilateral anterior shins. Similar non-tender nodules were present on the lower abdomen and lower back. There was no oral or ocular involvement. Skin punch biopsies were performed for further evaluation.

The patient underwent nerve conduction study for his paraesthesia in his hands which concluded that there was evidence of bilateral focal ulnar neuropathy at the elbow, that was moderate on the right and moderate-severe on the left. There was no evidence of focal median neuropathy on either side.

On day 4 of admission, the lip biopsy results returned, but the sample contained insufficient tissue for diagnostic purposes. The BioPlex Connective Tissue Disease (CTD) screen was also negative. At this point, the eGFR plateaued around 33 mL/min/1.73 m^2^ and corrected calcium remained high at 3.22 mmol/L. The renal team reviewed the patient and initiated amlodipine to optimise blood pressure control. Additionally, the patient received intravenous zoledronic acid for persistent hypercalcaemia, which had not responded to six days of fluid resuscitation.

**Figure 4:**
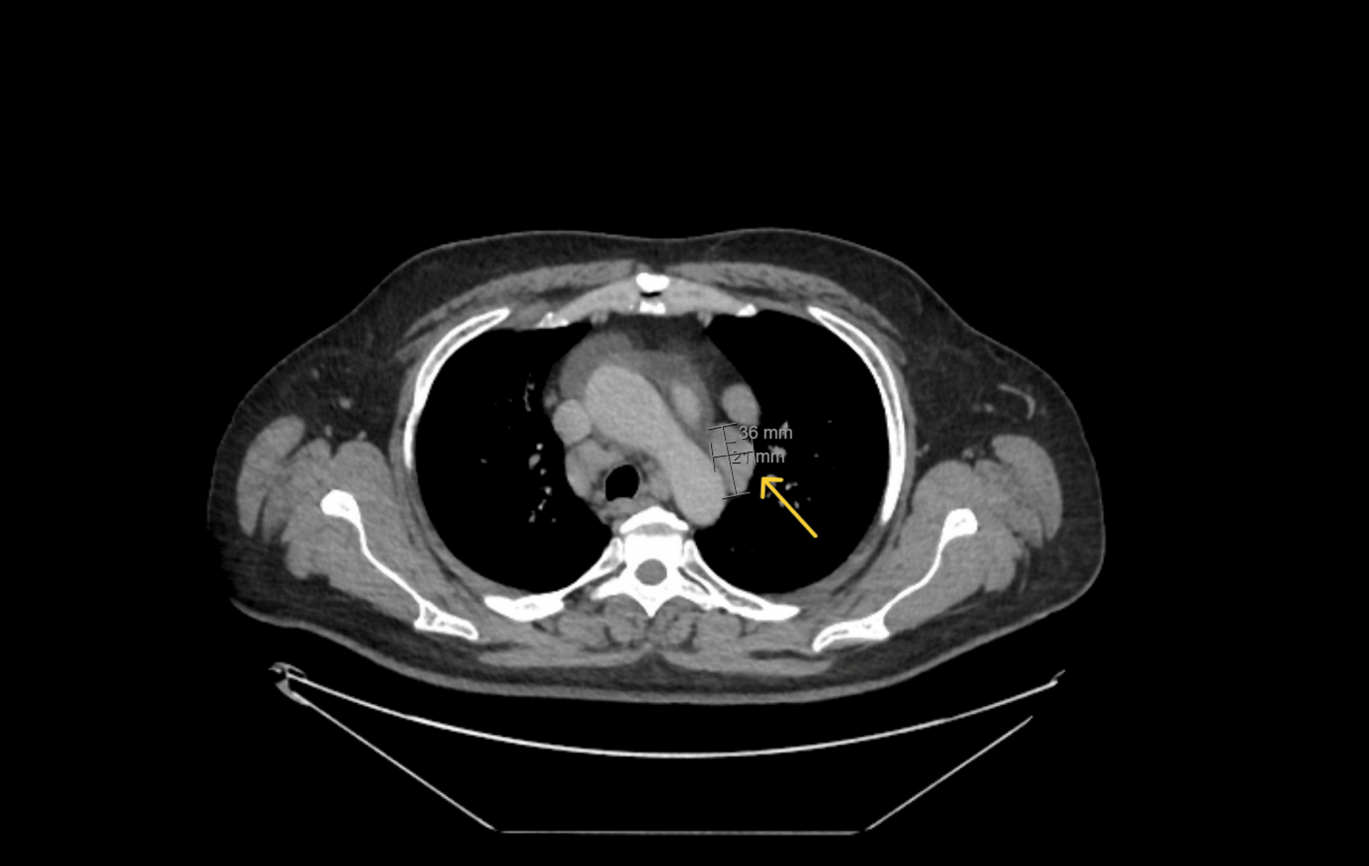
Arrow showing large lymph node in the aortopulmonary window.

**Figure 5:**
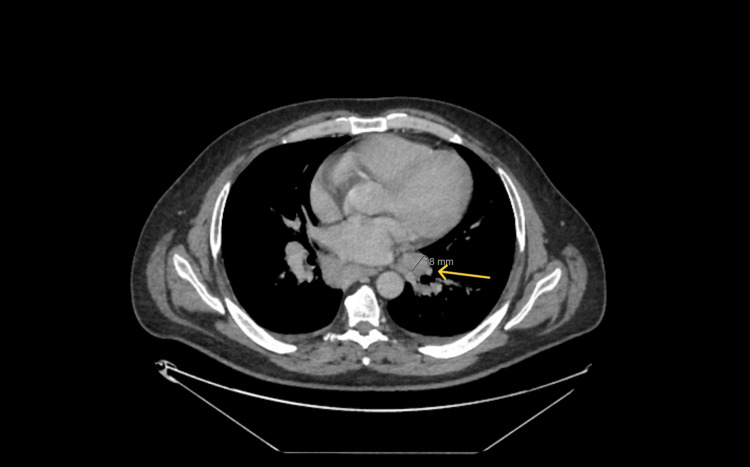
Arrow showing left inferior hilar node.

CT scan of the neck and CT TAP with contrast showed extensive mediastinal and hilar lymphadenopathy (Figures 4, 5), and the patient was referred to the respiratory clinic.

Skin biopsy revealed numerous focally confluent naked granulomata in the dermis extending into the subcutis. The histological findings indicated a sarcoid granulomatous inflammatory tissue reaction pattern. This confirmed the clinical diagnosis of sarcoidosis.

The patient was informed of the diagnosis of multi-system sarcoidosis with renal, eye, nerve and skin involvement. He was commenced on 60 mg prednisolone. Concurrently, he was prescribed omeprazole, alendronic acid, and co-trimoxazole. The patient was discharged on the same day of the skin biopsy results, with plans for follow-up in renal outpatient care. At the time of discharge, his corrected calcium level remained elevated at 3.22 mmol/L, creatinine was 193 umol/L and eGFR was 33 mL/min/1.73 m^2^.

Outcome and follow-up

The patient was reviewed ten days after his discharge date in the renal clinic, and he reported marked improvement of his overall symptoms. The calcium had improved to 2.58 mmol/L, creatinine to 126 umol/L and eGFR to 55 mL/min/1.73 m^2^.

One month after hospital admission, blood tests showed normal calcium of 2.38 mmol/L and eGFR >90 mL/min/1.73 m^2^. The management plan was to gradually wean off prednisolone, with regular outpatient reviews.

## Discussion

Primary hyperparathyroidism and malignancy account for the majority of hypercalcaemia cases [1,2]. However, less frequent etiologies such as granulomatous diseases can lead to elevated calcium levels through distinct pathophysiological mechanisms [1]. Sarcoidosis is the most well-recognised granulomatous cause but other granulomatous disorders such as tuberculosis, fungal infections (e.g., histoplasmosis), berylliosis, and certain lymphomas can also present with hypercalcaemia through similar mechanisms [2,3,5]. Awareness of these differentials is important for accurate diagnosis, especially when PTH levels are suppressed. This case highlights a diagnostic challenge where a patient with persistent hypercalcaemia and acute kidney injury was later confirmed to have sarcoidosis with inconclusive early findings and a low serum ACE level.

Sarcoidosis is a multisystem granulomatous disorder that can present with a wide variety of clinical symptoms depending on the organs involved [4]. Hypercalcaemia occurs in approximately 10-20% of sarcoidosis cases, making it a clinically significant clue in the diagnostic process [1,4]. The primary mechanism involves increased extrarenal activation of 1-alpha-hydroxylase by macrophages within granulomas, leading to elevated production of calcitriol (1,25-dihydroxyvitamin D), which in turn enhances intestinal calcium absorption [1,3,5]. Calcitriol also promotes bone resorption, further contributing to hypercalcaemia [1,3]. In rare cases, sarcoidosis may lead to humoral hypercalcaemia via parathyroid hormone-related protein (PTHrP) secretion [5]. This process is independent of parathyroid hormone (PTH) regulation, and a suppressed PTH in the context of elevated calcium is a key finding suggestive of a PTH-independent mechanism [1].

Serum ACE, an enzyme produced by granulomas, is commonly elevated in active sarcoidosis and used as a supportive diagnostic marker [6]. However, its diagnostic performance is limited: Kawai et al. reported a sensitivity of 41.4% and specificity of 89.9% at a cut-off value of 21.4 U/L [7]. Therefore, low or normal ACE levels, as observed in this patient, do not exclude sarcoidosis. The low ACE level in this case could be attributed to variability in disease activity or granuloma burden [8]. This emphasises the importance of clinical correlation which can be confirmed with histological evidence.

The clinical presentation in this patient was complex due to overlapping features of Sjögren’s syndrome and sarcoidosis, both of which can involve the salivary glands [9]. Initial parotid ultrasound findings of diffuse hypertrophy and heterogeneous echotexture in the salivary glands, along with sicca symptoms (dry eyes and mouth), suggested Sjögren’s syndrome [9]. However, the lack of specific antibodies and inconclusive lip biopsy necessitated further investigations.

The patient's systemic manifestations, such as significant weight loss, joint pain, a non-blanching lower limb rash, and neurological symptoms (ulnar nerve involvement), prompted consideration of a broader differential diagnosis. The CT findings of extensive mediastinal and hilar lymphadenopathy raised concerns for sarcoidosis, lymphoma, or tuberculosis. Histological confirmation of non-caseating granulomas on skin biopsy was ultimately what led to confirm the final diagnosis of sarcoidosis.

Systemic sarcoidosis is managed based on the severity of organ involvement. Corticosteroids are often the first-line treatment; they help reduce inflammation and can prevent disease progression [10]. When hypercalcaemia is present, corticosteroids also reduce extrarenal 1-alpha-hydroxylase activity, lowering calcitriol synthesis and intestinal calcium absorption [1,4,10]. This makes them the mainstay of treatment in hypercalcaemic patients. In cases of steroid-resistant or where a corticosteroid sparing strategy is required, alternative immunosuppressive agents such as methotrexate, azathioprine, leflunomide and hydroxychloroquine can be considered. When standard therapy fails, infliximab and other biologic agents are considered effective third-line drugs in sarcoidosis [11].

## Conclusions

This case underscores the complexity of evaluation of a patient with hypercalcaemia when faced with multiple systemic symptoms. The initial suspicion of Sjögren’s syndrome, based on imaging findings and sicca symptoms, was revised to sarcoidosis following a comprehensive clinical evaluation and histopathological confirmation. The presence of low serum ACE levels does not usually exclude the diagnosis, emphasising the importance of clinical suspicion and histopathological confirmation in such ambiguous cases. Sarcoidosis should be considered in patients with unexplained hypercalcaemia and systemic involvement, even when typical diagnostic markers are inconclusive.
